# l-asparaginase-based regimens followed by allogeneic hematopoietic stem cell transplantation improve outcomes in aggressive natural killer cell leukemia

**DOI:** 10.1186/s13045-016-0271-4

**Published:** 2016-04-18

**Authors:** Ki Sun Jung, Su-Hee Cho, Seok Jin Kim, Young Hyeh Ko, Eun-Suk Kang, Won Seog Kim

**Affiliations:** Division of Hematology-Oncology, Department of Internal Medicine, Samsung Medical Center, Sungkyunkwan University School of Medicine, Seoul, Korea; Division of Hematology-Oncology, Department of Internal Medicine, Pusan National University Yangsan Hospital, Pusan National University School of Medicine, Yangsan, Korea; Department of Pathology, Samsung Medical Center, Sungkyunkwan University School of Medicine, Seoul, Korea; Department of Laboratory Medicine and Genetics, Samsung Medical Center, Sungkyunkwan University School of Medicine, Seoul, Korea

**Keywords:** ANKL, l-asparaginase, Allogeneic HSCT, Response, Survival

## Abstract

Aggressive nature killer cell leukemia (ANKL) is a mature NK-T cell lymphoma with worse prognosis, but optimal treatment is unclear. Therefore, we analyzed the efficacy of l-asparaginase-based regimens for ANKL patients. Twenty-one patients who received dexamethasone, methotrexate, ifosfamide, l-asparaginase, and etoposide (SMILE) or etoposide, ifosfamide, dexamethasone, and l-asparaginase (VIDL) chemotherapy at Samsung Medical Center were selected. The overall response rate for all patients was 33 % (7/21); 38 % (5/13) in SMILE and 40 % (2/5) in VIDL, respectively. The median progression-free survival was 3.9 months (95 % CI 0.0–8.1 months) and median overall survival was 7.0 months (95 % CI 2.3–11.7 months). Treatment response (*P* = 0.001), hematopoietic stem cell transplantation (HSCT) (*P* = 0.007) and negative conversion of Epstein-Barr virus (EBV) DNA titer after treatment (*P* = 0.004) were significantly associated with survival. Thus, l-asparaginase-based regimens followed by allogeneic HSCT seem to improve the outcome for ANKL patients.

**Electronic supplementary material:**

The online version of this article (doi:10.1186/s13045-016-0271-4) contains supplementary material, which is available to authorized users.

## Findings

Aggressive natural killer cell leukemia (ANKL) is a rare lymphoma with aggressive clinical course [1, 2]. Treatment outcomes are worse and the median survival is less than 2 months [2–4]. However, optimal treatment of ANKL is not determined yet. Because the tumor cells of ANKL produce P-glycoproteins [5, 6], the treatment response to conventional chemotherapies such as cyclophosphamide, doxorubicin, vincristine, and prednisone (CHOP) is ineffective [4, 7, 8]. Instead, methotrexate and l-asparaginase, which are not affected by P-glycoprotein, are effective agents against NK-T cell lymphoma [3, 7, 9]. Therefore, we conducted this study to determine the efficacy of l-asparaginase-based regimes for patients with ANKL.

ANKL patients diagnosed by current World Health Organization classification who received dexamethasone, methotrexate, ifosfamide, l-asparaginase, and etoposide (SMILE) or etoposide, ifosfamide, dexamethasone, and l-asparaginase (VIDL) as first-line or second-line chemotherapy were enrolled from a lymphoma cohort at Samsung Medical Center between January 2008 and May 2015. We conducted in accordance with the Helsinki Declaration in our study, and our study was approved by the institutional review board of Samsung Medical Center. Clinical characteristics at diagnosis were analyzed for enrolled patients. The treatment response was evaluated followed by criteria that reported previous studies [2, 10]. We defined complete response as (1) improved laboratory findings (cytopenia and liver function tests) and symptoms (organomegaly and B symptoms), (2) no residual ANKL cells in bone marrow, and (3) no definite abnormalities in imaging including positron emission tomography or computed tomography. We defined partial response as improved laboratory findings, symptoms, and imaging but minimal residual ANKL cells in bone marrow. The Kaplan-Meier method was used for survival analysis, and the log-rank test was used to test differences in survival on univariate analysis.

A total of 21 patients were analyzed in this study. The characteristics for them are presented in Table 1. Thirteen patients (62 %) received SMILE as first-line chemotherapy. Of these patients, five patients showed response to SMILE, either complete response (*n* = 3, 23 %) or partial response (*n* = 2, 15 %). Thus, overall response rate (ORR) to SMILE as initial treatment was 38 %. Of patients who responded to SMILE, the three complete responders underwent allogeneic hematopoietic stem cell transplantation (HSCT) and were alive. Of the two partial responders, one received mitoxantrone, etoposide and cytarabine induction chemotherapy and is alive without relapse. The other patient underwent autologous HSCT but died from relapse. Five patients (24 %) were given VIDL as initial treatment. Of these patients, two exhibited complete response to VIDL chemotherapy and received allogeneic HSCT. Thus, the ORR to VIDL was 40 %. Of three patients (14 %) treated with SMILE as second-line chemotherapy, one died from sepsis and the other patients showed disease progression despite receiving full-dose SMILE. Thus, the ORR for all patients was 33 % (7/21). Treatment outcomes and characteristics of patients who received HSCT are in Additional files 1 and 2.

**Table 1:** Baseline patient characteristics (*N* = 21)

Characteristics	Number of patients	Percentage
Median age, years (range)	50 (16–75)	
Sex		
Male	14	67
Female	7	33
PS		
0–1	12	57
2–4	9	43
Initial presentation		
Fever	20	95
General weakness	1	5
DOE	1	5
Lt. neck swelling	1	5
B symptom (+)	20	95
Hepatomegaly	13	62
Splenomegaly	17	81
Pancytopenia	11	52
LFT abnormality	20	95
DIC	9	43
AKI	4	19
LDH > normal	20	95
Stage IV	21	100
IPI score		
Low	1	5
Low intermediate	7	33
High intermediate	7	33
High	6	29
Nodal involvement	7	33
Extranodal involvement	7	33
EBV ISH (+)	17	81
HLH feature	16	76
PET uptake at diagnosis	9	43

*PS* performance status, *DOE* dyspnea on exertion, *LFT* liver function test, *DIC* disseminated intravascular coagulopathy, *AKI* acute kidney injury, *LDH* lactate dehydrogenase, *IPI* international prognosis index, *EBV ISH* Epstein-Barr virus in situ hybridization, *HLH* hemophagocytic lymphohistiocytosis

**Fig 1:**
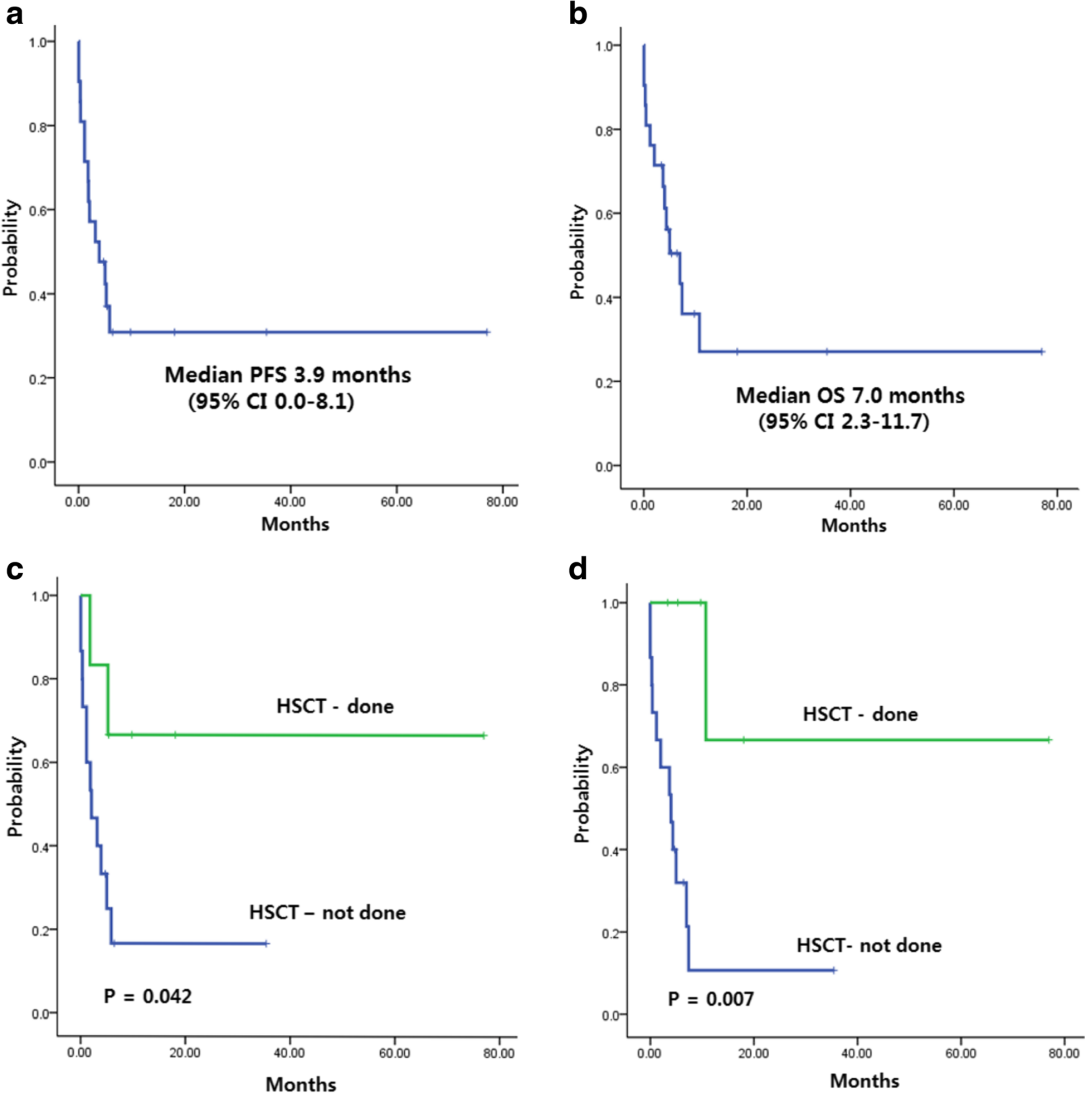
**a** Progression-free survival and **b** overall survival of 21 patients. **c**, **d** PFS and OS for patients who underwent HSCT and those who did not receive HSCT

The median progression-free survival (PFS) was 3.9 months (95 % CI 0.0–8.1 months, Fig. 1a) and median overall survival (OS) was 7.0 months (95 % CI 2.3–11.7 months, Fig. 1b). When univariate analysis for prognostic factors was performed, patients who received HSCT had a better OS (*P* = 0.007) and PFS (*P* = 0.042) than patients who did not undergo HSCT (Fig. 1c, d).

We also analyzed correlations between Epstein-Barr virus (EBV) DNA titers and survival (Additional file 3). EBV DNA titer at diagnosis (*P* = 0.311) and baseline EBV negativity (*P* = 0.307) were not associated with OS. Negativity of EBV DNA titer after treatment was significantly associated with OS (*P* = 0.004). But, no significant difference was seen in OS between the two subgroups according to change pattern of EBV DNA titer during treatment (*P* = 0.069). Clinical treatment response showed significant association with OS (*P* < 0.001).

In conclusion, early diagnosis and the use of an l-asparaginase-based regimen at initial diagnosis had promising efficacy for patients with ANKL. Also, allogeneic HSCT for responders to an l-asparaginase-based regimen might improve treatment outcomes for patients with ANKL.

## Additional files

Additional file 1: Summary of patient response and chemotherapy outcomes (A) SMILE as first-line chemotherapy. (A) Thirteen patients (62 %) received SMILE as first-line chemotherapy and 5 patients showed treatmentresponse. Thus, ORR was 38 %. (B) Five patients (24 %) underwent VIDL as first-line chemotherapy and 2 patients showed treatment response. Thus, ORR was 40 %. (C) Three patients (14 %) treated with SMILE as second-line chemotherapy but no responder showed. (DOCX 97 kb) Additional file 2: Summary of patients who underwent HSCT after SMILE or VIDL chemotherapy. One patient underwent autologous HSCT and 7 patients received allogeneic HSCT. No engraftment failure or treatment-related mortality occurred. But, 2 patients died from disease relapse (Autologous; 1, Allogeneic; 1). (DOCX 17 kb) Additional file 3: Survival according to EBV DNA titer and treatment response (A) Survival according to baseline EBV DNA. (A) Baseline EBV negativity did not affect OS (*p* = 0.307). (B) Negativity EBV DNA after treatment was significantly associated with OS (*p* = 0.004). (C) Change pattern (decreasing vs. increasing) of EBV DNA titer during treatment was not significantly associated with OS (*p* = 0.069). (D) Clinical treatment response showed significant association with OS (*p* < 0.001). (DOC 103 kb)

## Abbreviations

ANKL: aggressive nature killer cell leukemia
CHOP: cyclophosphamide, doxorubicin, vincristine, and prednisone
EBV: Epstein-Barr virus
HSCT: hematopoietic stem cell transplantation
ORR: overall response rate
OS: overall survival
PFS: progression-free survival
SMILE: dexamethasone, methotrexate, ifosfamide, l-asparaginase, and etoposide
VIDL: etoposide, ifosfamide, dexamethasone, and l-asparaginase
