# Supplementary material for: l-asparaginase-based regimens followed by allogeneic hematopoietic stem cell transplantation improve outcomes in aggressive natural killer cell leukemia
Source: J Hematol Oncol. 2016 Apr 18;9:41. doi: 10.1186/s13045-016-0271-4 (PMC4835915; doi:10.1186/s13045-016-0271-4)

**Supplementary File 1**. Summary of patient response and chemotherapy outcomes

(A) SMILE as first-line chemotherapy


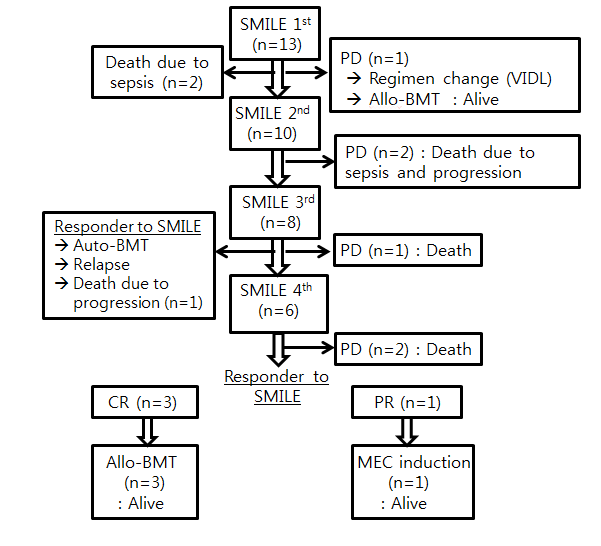


(B) VIDL as first-line chemotherapy


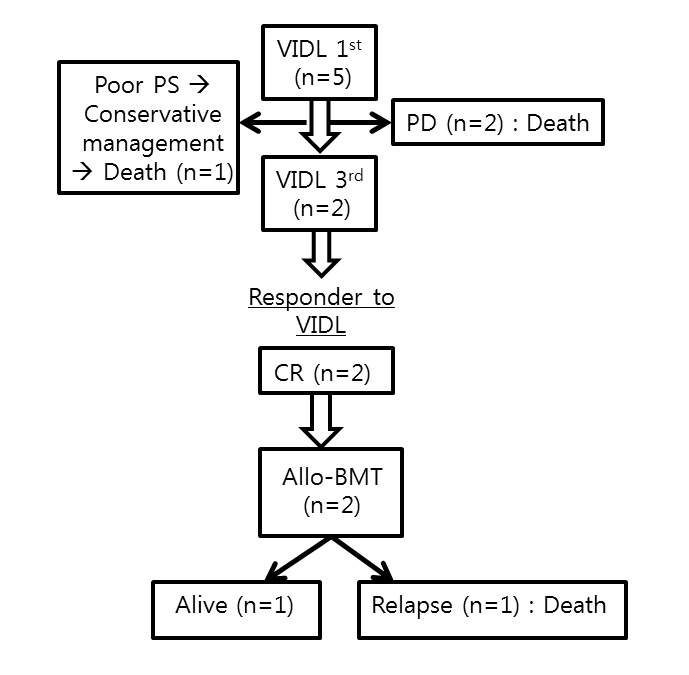


C) SMILE as second-line chemotherapy


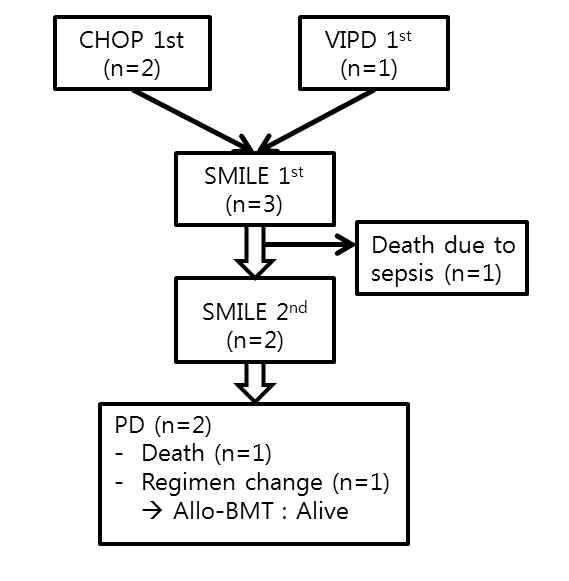

Supplement: Additional file 1: — Summary of patient response and chemotherapy outcomes (A) SMILE as first-line chemotherapy. (A) Thirteen patients (62 %) received SMILE as first-line chemotherapy and 5 patients showed treatmentresponse. Thus, ORR was 38 %. (B) Five patients (24 %) underwent VIDL as first-line chemotherapy and 2 patients showed treatment response. Thus, ORR was 40 %. (C) Three patients (14 %) treated with SMILE as second-line chemotherapy but no responder showed. (DOCX 97 kb) [file 13045_2016_271_MOESM1_ESM.docx]
