# Supplementary material for: l-asparaginase-based regimens followed by allogeneic hematopoietic stem cell transplantation improve outcomes in aggressive natural killer cell leukemia
Source: J Hematol Oncol. 2016 Apr 18;9:41. doi: 10.1186/s13045-016-0271-4 (PMC4835915; doi:10.1186/s13045-016-0271-4)
Supplement: Additional file 2: — Summary of patients who underwent HSCT after SMILE or VIDL chemotherapy. One patient underwent autologous HSCT and 7 patients received allogeneic HSCT. No engraftment failure or treatment-related mortality occurred. But, 2 patients died from disease relapse (Autologous; 1, Allogeneic; 1). (DOCX 17 kb) [file 13045_2016_271_MOESM2_ESM.docx]

**Supplementary File 2.** Summary of patients who underwent HSCT after SMILE or VIDL chemotherapy.

| Characteristics at diagnosis | | | | |  | SMILE or VIDL | | |  | HSCT | |  | Post-HSCT | Last follow up status |
| --- | --- | --- | --- | --- | --- | --- | --- | --- | --- | --- | --- | --- | --- | --- |
| S/A | LDH | B Sx | EBV titer | IPI |  | Regimen | Number of cycles | Response |  | Type | Conditioning regimen |  | EBV titer |  |
| M/16  M/28  M/36  M/56  M/34  F/61  M/49  F/55 | N  H  H  H  H  H  H  H | +  +  +  +  +  +  +  - | -  286  10264  1840  1481  33.5  25  7055 | L  HI  HI  LI  H  HI  LI  LI |  | SMILE  SMILE  VIDL  SMILE  SMILE  VIDL  SMILE🡪VIDL  SMILE 🡪  GDP/L-aspa | 3  4  3  4  4  3  1🡪1  2🡪3 | PR  CR  CR  CR  CR  CR  PD 🡪 PD  PD 🡪 PD |  | Auto  Allo  Allo  Allo  Allo  Allo  Allo  Allo | Bu-Cy-VP16  FluBu4/rATG/TBI  FluBu4/rATG  FluBu4/rATG  FluBu4/rATG/TBI  FluBu2/rATG  FluBu4/rATG  FluBu4/rATG |  | -  2  -  -  -  757.28  0.57  - | Died  Alive  Alive  Alive  Alive  Died  Alive  Alive |

N, normal; H, high; +, presence; -, none; EBV titer: copies/ml; S, sex; A, age; LDH, lactate dehydrogenase; Sx, symptoms; IPI, international prognostic index; L, low; LI, low-intermediate; HI, high-intermediate; H, high; CR, complete response; PR, partial response; Bu-Cy-VP16, busulfan, cyclophosphamide, etoposide; FluBu4/rATG, fludarabine, busulfan, rATG; TBI, total body irradiation
