# Supplementary material for: l-asparaginase-based regimens followed by allogeneic hematopoietic stem cell transplantation improve outcomes in aggressive natural killer cell leukemia
Source: J Hematol Oncol. 2016 Apr 18;9:41. doi: 10.1186/s13045-016-0271-4 (PMC4835915; doi:10.1186/s13045-016-0271-4)

**Supplementary File 3.** Survival according to EBV DNA titer and treatment response

(A) Survival according to baseline EBV DNA.


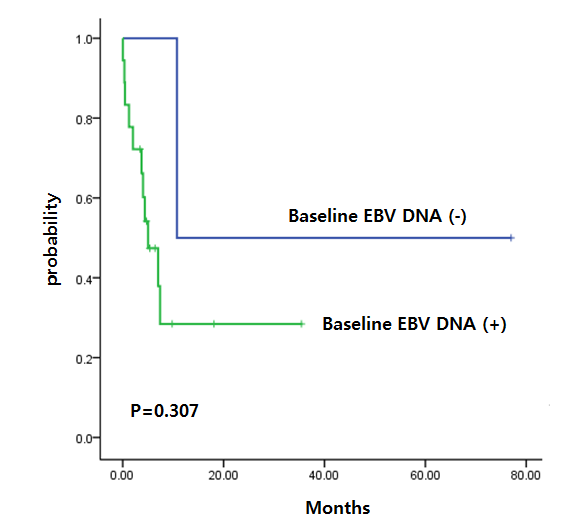


(B) Relationship between EBV DNA negativity after treatment and OS.


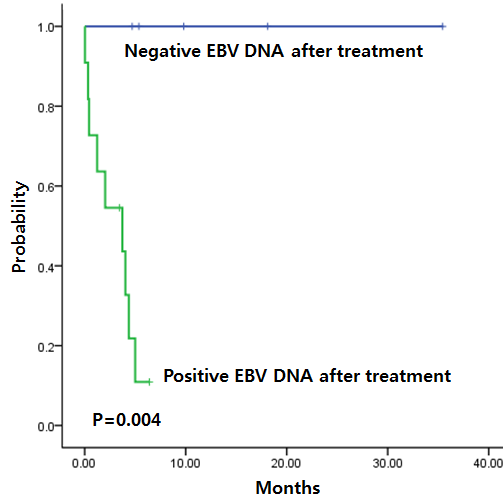


(C) Relationship between change in EBV DNA after treatment and OS.


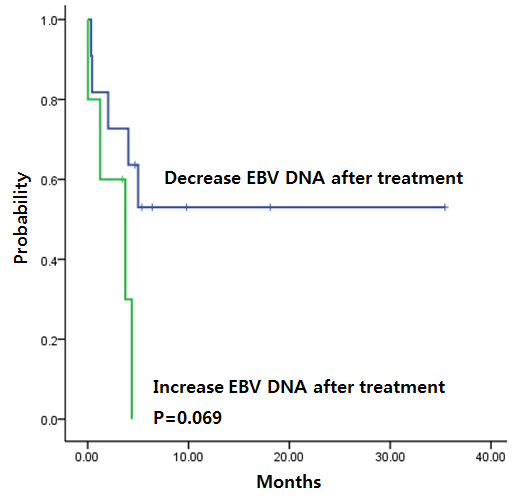


(D) Relationship between treatment response and OS.


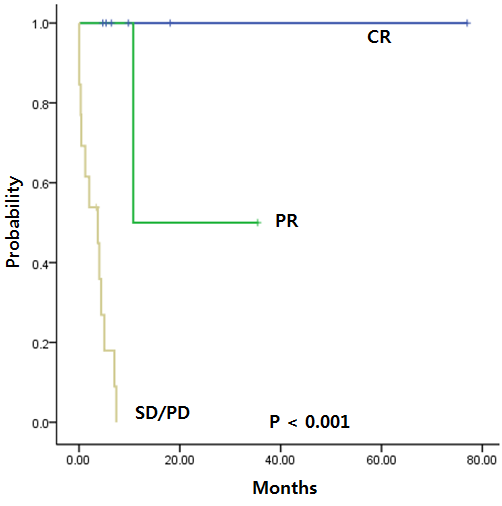

Supplement: Additional file 3: — Survival according to EBV DNA titer and treatment response (A) Survival according to baseline EBV DNA. (A) Baseline EBV negativity did not affect OS (p = 0.307). (B) Negativity EBV DNA after treatment was significantly associated with OS (p = 0.004). (C) Change pattern (decreasing vs. increasing) of EBV DNA titer during treatment was not significantly associated with OS (p = 0.069). (D) Clinical treatment response showed significant association with OS (p < 0.001). (DOC 103 kb) [file 13045_2016_271_MOESM3_ESM.doc]
